# Mitigation of ammonia volatilization and nitrate leaching via loss control urea triggered H-bond forces

**DOI:** 10.1038/s41598-019-51566-2

**Published:** 2019-10-22

**Authors:** Zhipan Ma, Yanjun Yue, Mengxi Feng, Yushun Li, Xue Ma, Xu Zhao, Shenqiang Wang

**Affiliations:** 1 State Key Laboratory of Soil and Sustainable Agriculture, Changshu National Agro-Ecosystem Observation and Research Station, Institute of Soil Science, Chinese Academy of Sciences, Nanjing, 210008 China; 2 Henan Xin Lian Xin Fertilizer Co., Ltd., Xinxiang, 453700 China

**Keywords:** Environmental impact, Materials science

## Abstract

Excess nitrogen (N) fertilizer applied to crops, which discharges to the environment, principally through denitrification, runoff, leaching, and volatilization, results in a waste of resources and pollution. Here, a high-performance loss control urea (LCU) was prepared by adding a loss control agent (LCA) with high thermal stability, large specific surface area, and good water retention capacity complex (6%) to traditional urea (94%). The existence of hydrogen bonds between LCA and N source for LCU in the presence of water enhanced N source adsorption capacity, where adsorption between LCA and NH_4_^+^-N was strongest, for urea and NO_3_^−^-N was weakest. In a laboratory experiment, cumulative losses of NH_3_ volatilization from soils treated with N application rates of 80, 160 and 240 kg N ha^−1^ were 14.8, 18.1, and 24.2% for urea, respectively, and 10.1, 12.7, and 17.5% for LCU. Simulated rapid and long-term leaching experiments showed that, compared with urea, LCU reduced N leaching loss within 30 d, and delayed long-term N leaching loss. Hydrogen bonds in LCU effectively controlled NH_3_ volatilization and N leaching loss. This type of LCU may optimize supply of N in soils and increase adsorption and utilization of N in crops.

## Introduction

Over 100 million tonnes of N fertilizer is applied annually to agricultural crops worldwide; however, in the last 30 years, the N use efficiency in cereals of China is c. 27–40%^1–3^, and much of the surplus N that is lost to the environment results in atmospheric and freshwater pollution^4^. Therefore, sustainable agriculture depends on achieving reductions in N inputs, while maintaining high levels of crop yield. Approaches that address this challenge tend to be at the agro-economic scale and include optimization of crop rotations, environment of sensor-based fertilizer applications, integrated crop management that uses livestock systems^5,6^, and improvement of crop N use efficiency^7^. Among these, improvement of N use efficiency is key and has been widely studied. N fertilizer not utilized by crops tends to discharge to the environment through a range of processes that include nitrification-denitrification, ammonia (NH_3_) volatilization, leaching, and runoff^8^. In addition to traditional approaches designed to improve N use efficiency, such as use of appropriate rates and deep applications of N, current techniques include site-specific and real-time N and nutrient management, non-destructive quick testing of plant N status, and application of controlled/slow release fertilizers^9^; that latter approach is considered to be the quickest and most convenient for reducing fertilizer losses and increasing fertilizer use efficiency^10^.

Reduction of NH_3_ volatilization and leaching losses is key in the improvement of N use efficiency, and the employment of controlled/slow release N fertilizer mainly tends to delay its availability for plant uptake and use after application, or extend its availability to the plant significantly longer than a reference ‘rapidly available nutrient fertilizer’ through a specific control mechanism^11^. For example, the coated N fertilizer control N interactions between crops and soils by delaying availability release; the urease inhibitor which inhibits hydrolytic action on urea by the enzyme urease; and the nitrification inhibitor which inhibits the biological oxidation of ammoniacal-N to nitrate-N. In addition, other methods tend to enhance N adsorption through the addition of high adsorption capacity substances. Moreover, The effect of controlling NH_3_ volatilization after using them, as stated earlier^12^ that the use of non-urea based fertilizers (ammonium nitrate and ammonium sulfate), urease inhibitors, controlled release fertilizers, deep placement of fertilizers, irrigation, and addition of mixed amendments (pyrite, zeolite, and organic acids) decreased NH_3_ volatilization by 75, 54, 68, 55, 35 and 35%, respectively. Use of ammonium nitrate or ammonium sulfate may contribute to soil acidification and lead to subsequent decreases in NH_3_ volatilization^13–15^, but long-term application may result in significant damage to the soil; therefore, the use of controlled release fertilizers may represent an effective and convenient approach to reduce NH_3_ volatilization. Sanchita Mandal^16^ and Chen *et al*.^17^ found that addition of biochar to soils reduced NH_3_ volatilization by approximately 70%^18–20^, where mitigation of N leaching losses following biochar addition is mainly attributed to NH_4_^+^ adsorption and enhanced N immobilization^21–23^, whereas the high financial cost of biochar limits its extensive use. While application of nitrification inhibitors mitigates NO_3_^−^-N leaching loss^24–26^, it may increase the loss of NH_3_ volatilization, influence yield and quality of partial crops and alter physicochemical properties and biological activity in soils^27^. Although N leaching losses from soils have been found to be prevented by trees as ecosystem service providers^28^, optimization of timing of tillage^29^, combination of low quality maize stover residue with N fertilizer^30^, and reductions in N application rates^31,32^, the efficacy of these measures is production system-dependent.

Peng *et al*.^33^ reduced N leaching losses through the use of controlled-release N fertilizer that have been developed during the past 20 years using chemical or physical techniques, such as sulfur coated, urease inhibitor, urea formaldehyde and resin coated^34–36^, and among them, the employment of the resin coating type is the most stable and extensive. Most types of controlled release coated fertilizer are encapsulated by a slow degrading polymer^37–39^ or materials that have strong adsorption properties^40,41^, but although these products effectively enhance N use efficiency, the problem of migration of decomposed N to crops remains unresolved. A recently developed type of fertilizer, known as loss control fertilizer (LCF), effectively reduces NH_3_ volatilization and leaching loss^42–45^ as a result of added attapulgite that is modified by high-energy electron beam irradiation and treated with O_3_ oxidation and hydrothermal processes^46^. The lack of need for polymer coating materials in LCFs during the production process facilitates single field applications and confers environmentally sustainable characteristics, such as natural degradation, so that their long-term application may improve soil physical and chemical properties, soil fertility and water retention capacity^45^, crop biological traits, and crop yield, while reducing N loss that contribute to improved N utilization rates^47–54^. However, the lack of understanding of underlying mechanisms associated with these positive effects of LCFs^55^ limits their potential use. In this study, we investigated effects of composition, performance, and associated mechanisms of N losses, measured as NH_3_ volatilization and nitrate leaching, of a loss control urea (LCU)^56^ as a type of LCF that contained silica aluminate mineral of montmorillonite as a loss control agent (LCA)^57^.

## Results and Discussion

### Adsorption of sources of N

**Figure 1:**
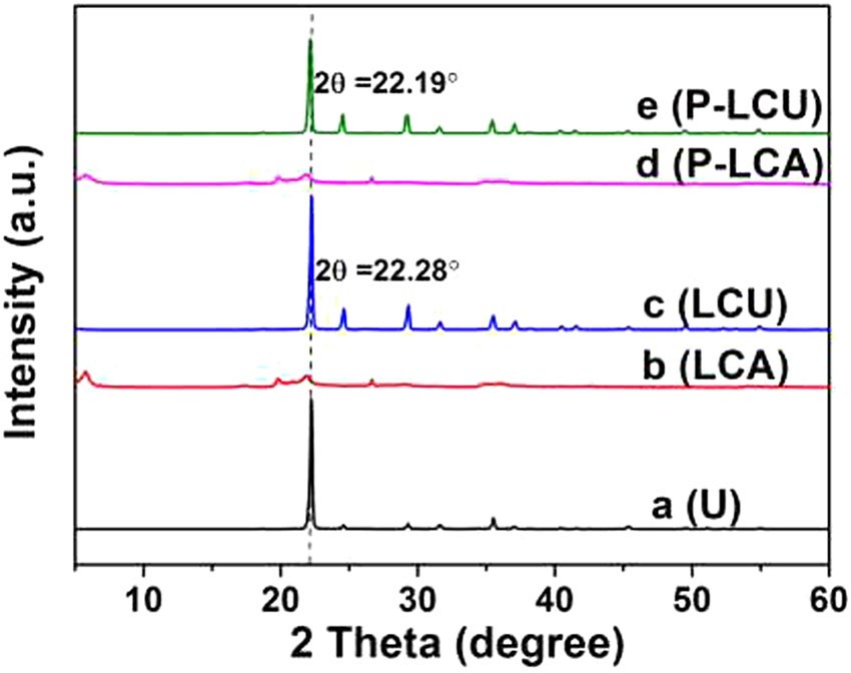
XRD patterns of (**a**) commercial urea, (**b**) loss control agent, (**c**) loss control urea, (**d**) pretreatment loss control agent, and (**e**) pretreatment loss control urea.

We used XRD to analyze the structure and composition of urea (U), LCA, and LCU, and found LCA was the silica aluminate mineral of montmorillonite, while comparison of LCA and LCU standard peaks showed that LCU comprised 94% urea and 6% LCA (Table 1), moreover, the elemental analyses of LCU obtained by XRF (Supplementary Table S1) are in well agreement with the result from XRD, further revealing the component content of the LCU; comparison of LCU with P-LCU showed that LCU was a simple physical mix of urea and LCA (Fig. 1). These results demonstrate that part of the urea particle enters the channel of P-LCU^58^. Analysis of the mass ratio of LCA and urea in LCU showed that LCA interacted with type of N source in the LCU in the presence of water, because we showed that a specified amount of LCA adsorbed urea-N and NH_4_^+^-N from NH_4_Cl, and NO_3_^−^-N from NaNO_3_ at a range of concentrations (0.1, 0.01, 0.005, and 0.002 mol L^−1^). Thus, it is possible to quantify adsorption capacity of LCA to N source types. We used two approaches to calculate the amount of adsorbed N by the LCA: firstly, concentration of N source following adsorption by LCA was assumed to approximate the adsorption capacity of LCA, and secondly, we used elemental analysis of N from dried LCA to directly measure the adsorbed N content by LCA. Results from the two approaches were generally consistent and variation was within the acceptable range (Table 2): LCA mainly adsorbed urea-N and NH_4_^+^-N, but adsorbed low levels of NO_3_^−^-N, where adsorption of NH_4_^+^-N was 1.6-fold greater than urea-N, and 3-fold greater than NO_3_^−^-N. Therefore, the adsorption capacity between LCA and NH_4_^+^-N was the strongest, while that between urea-N and NO_3_^−^-N was weakest under the presence of water.

**Table 1:** Relative content (wt.%) of components of loss control agent and loss control urea.

Sample	Montmorillonite (wt.%)	Cristobalite (wt.%)	Quartz (wt.%)	Urea (wt.%)
LCA	82	13	5	—
LCU	6	—	—	94

**Table 2:** Adsorption capacity of LCA on urea-N, NH_4_^+^-N and NO_3_^−^-N concentration.

N source	LCA	Solution A	Solution B	A-B	N amount^a^	N amount^b^
	mg	mg N/L	mg N/L	mg N/L	mg N/mg LCA	mg N/mg LCA
urea-N0.1 mol/L	50	1414	1089	325	1.44	1.24
urea-N0.01 mol/L	5	141	118	23	1.01	0.86
urea-N0.005 mol/L	2.5	70.5	60.1	10.4	0.92	0.79
urea-N0.002 mol/L	1	28.2	24.5	3.7	0.81	0.68
NH_4_^+^-N 0.1 mol/L	50	1414	914	500	2.22	1.93
NH_4_^+^-N 0.01 mol/L	5	141	107	34	1.54	1.22
NH_4_^+^-N0.005 mol/L	2.5	70.5	53.5	17	1.5	1.11
NH_4_^+^-N0.002 mol/L	1	28.2	22	6.2	1.38	1.02
NO_3_^−^-N0.1 mol/L	50	1414	1270	144	0.63	0.49
NO_3_^−^-N0.01 mol/L	5	141	129	12	0.52	0.37
NO_3_^−^-N0.005 mol/L	2.5	70.5	65.5	5	0.44	0.31
NO_3_^−^-N0.002 mol/L	1	28.2	26.6	1.6	0.36	0.12

Solution A: initial concentration of nitrogen; Solution B: concentration after adsorption by LCA; N amount values are calculated from ^a^concentration gradient and ^b^elemental analysis.

### Mechanisms of N loss

**Figure 2:**
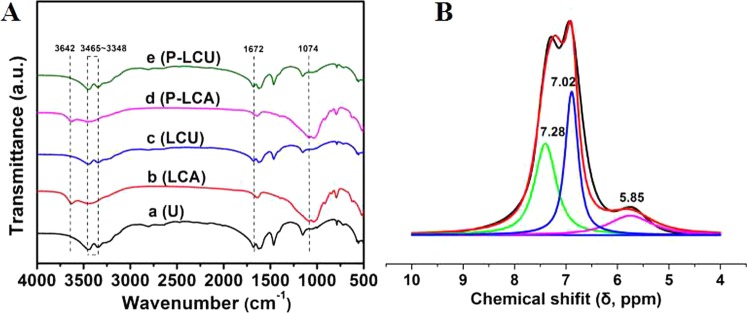
(**A**) FT-IR spectra of (a) commercial urea, (b) loss control agent, (c) loss control urea, (d) pretreatment loss control agent, (e) pretreatment loss control urea. (**B**) ^1^H MAS NMR spectra of pretreatment loss control urea.

We analyzed the force between LCA and N source using FTIR and ^1^H MAS NMR, where vibrational bands for commercial urea, LCU, and P-LCU (Fig. 2A, curves a, c, and e, respectively) were in ranges of 3348–3465 and 1672 cm^−1^, showing they belonged separately to the –CONH_2_ and –NH_2_ groups^59^. Content of LCA was very low in LCU, as shown by obscure peaks that indicate that LCU preparation was a simple physical mix of the LCA and urea. The LCA and P-LCA (Fig. 2A, curves b and d, respectively) showed separate vibrational bands for the –SO_2_ and –OH groups at 1074 and 3642 cm^−1^, indicating they are highly susceptible to hydrogen bonds with sources of N and water molecules. We also used ^1^H MAS NMR spectra of P-LCU to determine the existence of a hydrogen bond and the deconvolution of ^1^H MAS NMR by fitting with multiple peaks is shown in Fig. 2B. Therein, the peak of 7.28 ppm (35%) was probably attributed to the hydrogen bond formed between LCA and urea or LCA and NH_4_^+^-N, while the peak of 7.02 ppm (47%) one without H-bond^46,60^. Therefore, in addition to van der Waals forces, there was formation of hydrogen bonds between LCA and urea in the presence of water that increased adsorption capacity of LCA.

### LCU physicochemical properties

**Figure 3:**
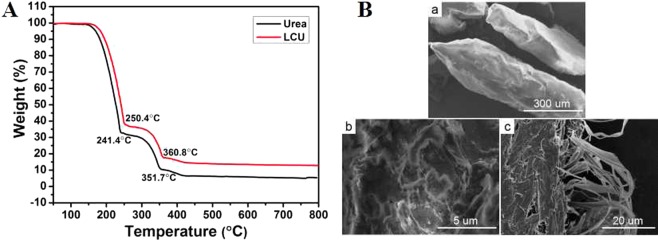
(**A**) TG curves of urea and loss control urea (LCU) samples. (**B**) SEM images of (a) commercial urea, (b) loss control urea and (c) pretreatment loss control urea.

TG curves of urea and LCU (Fig. 3A) showed a shift in mass loss of LCU between c. 240 and 350 °C to between c. 250 and 360 °C, indicating that LCA effectively enhanced the thermal stability of urea^46^ as a result of greater stability of LCU than urea conferred by the effects of hydrogen bonding. SEM images of urea, LCU, and P-LCU (Fig. 3B) showed a stone-like, smooth surface morphology of urea, a rough surface of LCU, and a contrasting micro/nano network surface of P-LCU. The surface morphologies of LCU and P-LCU are attributed to the addition of LCA that had a morphology of agglomerated particles (Supplementary Fig. S1a); the surface of P-LCA comprised uniformly dispersed pores (Supplementary Fig. S1b). These results suggest that, following hydrothermal pretreatment, LCU creates a diffuse surface with more network channels that facilitates adsorption of urea and reduces its loss.

**Figure 4:**
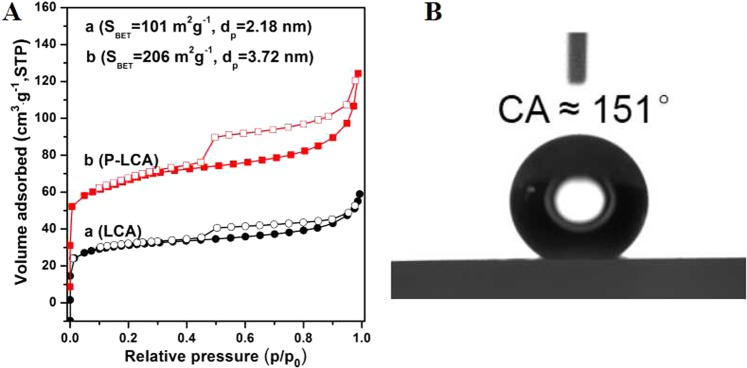
(**A**) N_2_ adsorption-desorption isotherm of (a) loss control agent and (b) pretreatment loss control agent. (**B**) Contact angle of water droplets on the surface of loss control agent.

N_2_ sorption isotherms of LCA and P-LCA (Fig. 4A) showed they were type I at low relative pressure (P/P_0_ < 0.1), with an intrinsic microporous structure, while they were type IV at high relative pressure (P/P_0_ > 0.6) due to a mesoporous structure. We found that surface area and pore diameter of LCA were 101 m^2^ g^−1^ and 2.18 nm, respectively, while P-LCA had a surface area of 206 m^2^ g^−1^ and pore diameter of 3.72 nm, indicating that hydrothermal pretreatment improved pore formation and associated adsorption performance. This result supports previous indications from SEM characterizations, and shows that when LCA adsorbed water, its surface become viscous and hydrophobic.

We investigated this hydrophilic-hydrophobic property of LCA using contact-angle assessment (Fig. 4B). If a water droplet contacts LCA, it yields a contact angle of up to 151° that indicates super hydrophobicity^61^, such that small amounts of adsorbed water may saturate LCA. We assessed water retention capacity of LCA (Supplementary Fig. S2), where LCA continuously adsorbed water for 3 h, and after 24 h of filtration, the moisture content of LCA reached 204%; its moisture content remained high (199%) after storage for 7 d in ambient conditions. These results show high water retention performance of LCA, and indicate that when LCA is mixed with urea, sources of N may be stored in the surface pores following contact with water due to an increased hydrophobic status of the surface that prevents further absorption of water.

### LCU resistance to N loss

**Figure 5:**
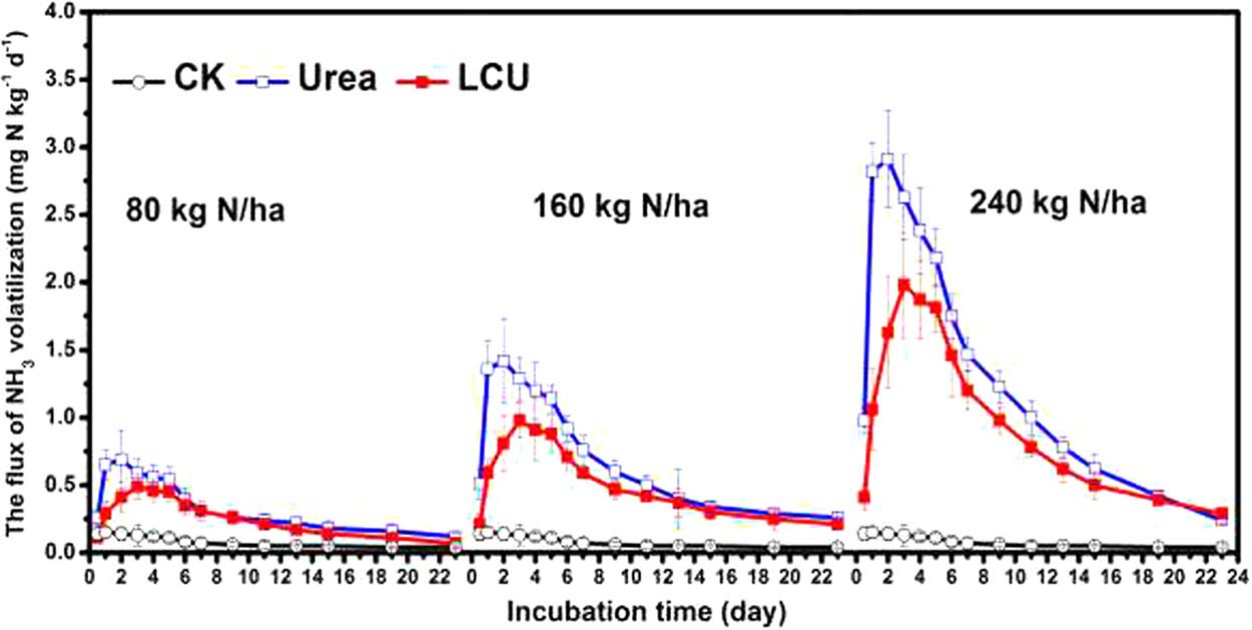
The time course of NH_3_ volatilization rate from a 23-day soil incubation treated with urea or LCU at N rates of 0, 80, 160 and 240 kg N ha^−1^ under 30 °C and 65% soil water-holding capacity. Error bars indicate standard deviation of replicates (n = 3).

We simulated NH_3_ volatilization, leaching, and runoff, based on this principal N loss pathway and observed the resistance control performance of LCU to N loss. Fluxes in NH_3_ volatilization from soil treated with urea reached a maximum 2 d after application at three levels of N (80, 160, and 240 kg N ha^−1^), where they ranged between 0.69 and 2.91 mg N/(kg·d); however, fluxes from soil treated with LCU reached a maximum 3 d after application, where they were in the range of 0.49–1.99 mg N/(kg·d) (Fig. 5). Fluxes in NH_3_ volatilization tended to remain stable from the 9 d after treatment, when N was applied at 80 kg N ha^−1^; when N application rates were 160 and 240 kg N ha^−1^, fluxes tended to stabilize at 11 and 15 d, respectively. Fluxes in NH_3_ volatilization were consistently lower for LCU than urea, regardless of N application rate, indicating that application of LCU may reduce NH_3_ volatilization. Cumulative losses of NH_3_ volatilization from soils treated with LCU were lower than from urea treatment under the same levels of N application (Supplementary Fig. S3): when N application rates were 80, 160 and 240 kg N ha^−1^, cumulative losses from soils treated with urea and LCU were 14.8% and 10.1%, 18.1% and 12.7%, and 24.2 and 17.5%, respectively. These results, combined with the previous characterizations, indicate it is likely that the hydrogen bond adsorption force of LCU between LCA and N sources reduced fluxes in NH_3_ volatilization and cumulative losses from the soils that were also reduced with decreasing N application rate.

Soils in drought areas tend to be sandy, and under these conditions, fertilizer may easily be leached with irrigation and rainwater. We assessed effect of LCU on leaching loss of N, and found that mass percent of retained TN in sand was 62.7% and 75.5% for urea and LCU, respectively, and mass percent of TN leaching loss was 23.1% and 11.6%, respectively, indicating LCU was more effective in leaching loss control than urea (Supplementary Fig. S4). Moreover, the mineral nitrogen content of leachate and sand retention (Supplementary Fig. S5) indicated that N leaching loss was mainly based on NO_3_^−^-N both for urea and LCU in the rapid N leaching experiments. And compared with traditional urea, the employment of LCU could obviously retain more NH_4_^+^-N and delay nitrification as a result of the presence of hydrogen bonds between LCA and NH_4_^+^-N.

**Figure 6:**
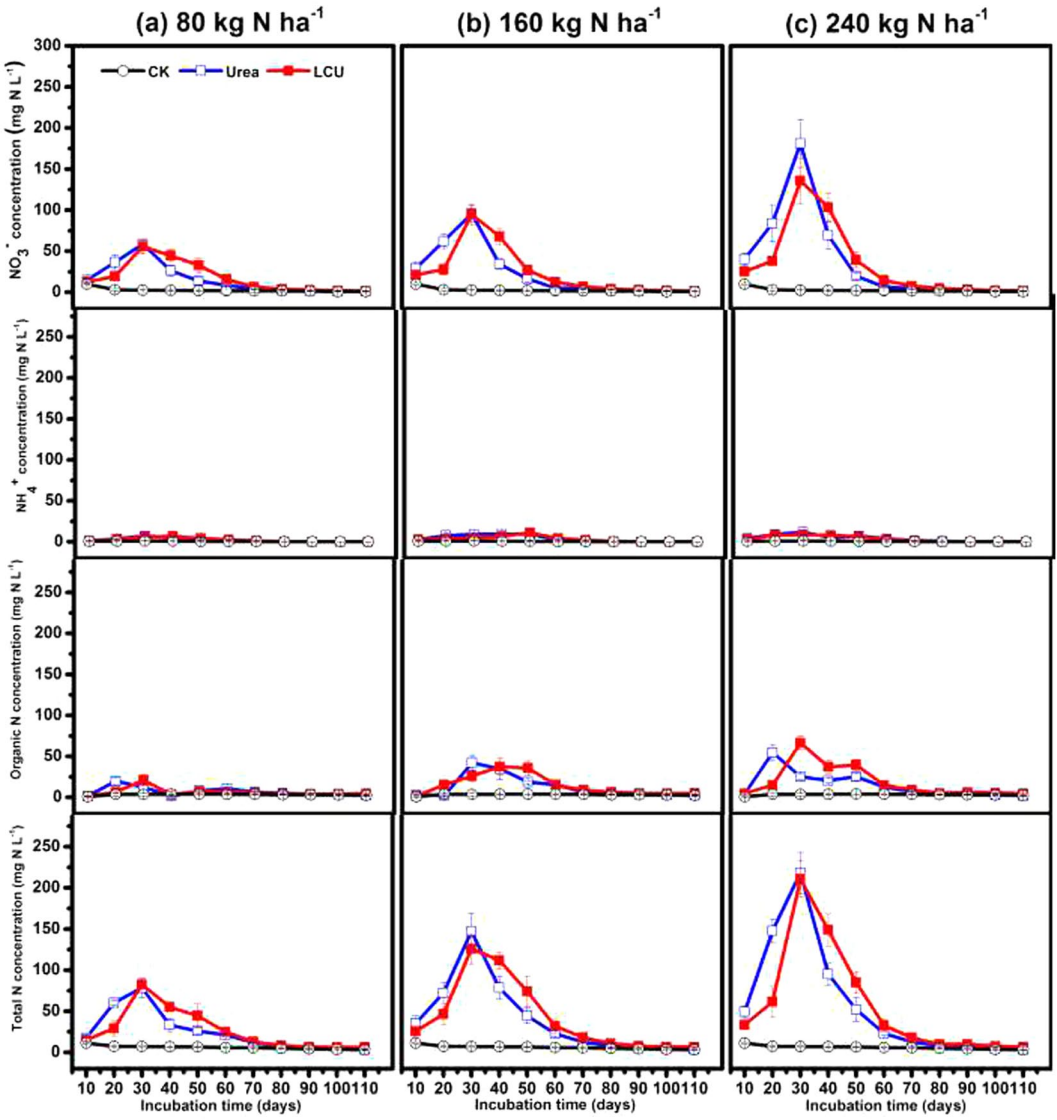
The time course of nitrogen (N) concentration in leachates from a 110-day soil leaching incubation treated with urea or LCU at N rates of 0, 80 (**a**), 160 (**b**) and 240 (**c**) kg N ha^−1^ under 30 °C and 65% soil water-holding capacity. Organic N (ON) was calculated as the differences between total N, NO_3_^−^-N and NH_4_^+^-N. Error bars indicate standard deviation of replicates (n = 3).

We assessed the long-term resistance control performance of LCU to N loss, by measuring the time course of N (NO_3_^−^-N, NH_4_^+^-N, Organic N and Total N) concentration of leachate from soil column over 110 d (Fig. 6). We found that when urea was applied with one of three levels of N (80, 160, and 240 kg N ha^−1^), TN concentration of leachate peaked 30 d after application, before reaching levels associated with no treatment after 80 d. In contrast, TN concentration in LCU was lower than for conventional urea up to 30 d after treatment, and higher between 40 and 70 d after treatment. Leachate NO_3_^−^-N concentration curves were similar to the corresponding TN at the three levels of N for LCU and urea, indicating that N leaching loss was mainly based on NO_3_^−^-N. Leaching loss of urea was greater than that of LCU in the first 30 d after treatment; this result corresponds to those for the rapid leaching test reported above. However, after 40 d, leaching loss of LCU was greater than that of urea, indicating that LCU may delay longer term leaching loss of N. However, for both LCU and urea, NH_4_^+^-N concentration of leachate was generally low, and there was little difference between the two types of fertilizer. However, the organic N concentration curves of the leachate were different between urea and LCU treatment. As the figure showed that the peak time of the latter (LCU) is obviously 10–20 days later than the former (urea). Moreover, We found there was no significant difference (except that LCU treatment had slightly higher nitrogen retention than urea treatment) in the amount of leaching and retention of fertilizer N at the end of experiment from 110 days incubation between conventional urea and LCU treatment among all levels of N addition (Supplementary Fig. S6), indicating that under strong leaching conditions without crop growth, applied LCU had no effects on N leaching loss. However, Supplementary Fig. S7 give the amounts of retention of fertilizer nitrogen (N) respectively within the top, middle and bottom of the soil column at the end of strong leaching experiment from 110 days incubation. We could get that the soil column with LCU treatment obviously retained more fertilizer N amount in the middle and upper layers than using urea, and the N amount in the soil column for employing urea was mainly left in the bottom layer. The results indicated that the application of LCU could mitigate the increase of N concentration in the bottom layer of the soil column to some extent.

Overall, our results show that LCU may retain nutrients shortly after application of fertilizer that may delay losses of fertilizer N caused by excessive applications; this may optimize supply of N in the soil and increase adsorption and utilization of N in crops.

## Conclusions

A high-performance loss control urea (LCU) prepared by mechanically mixing 6% of loss control agent (LCA), which mainly comprised montmorillonite, with 94% of urea. The LCU exhibited higher thermal stability, larger specific surface area, and better water retention capacity compared to traditional urea following hydrothermal treatment. For hydrothermal treated LCU, hydrogen bonds between LCA and N source (especially NH_4_^+^-N) reduced loss of N. Cumulative losses of NH_3_ volatilization from soils treated with LCU decreased by 4.7%, 5.4%, and 6.7% compared with traditional urea at N application rates of 80, 160 and 240 kg N ha^−1^, respectively. Compared with traditional urea, LCU reduced short-term N leaching following application of N fertilizer and delayed long-term nitrification. Our study shows that use of LCU may effectively control NH_3_ volatilization and N leaching loss, principally as a result of the presence of hydrogen bonds between LCA and NH_4_^+^-N. We conclude that the use of LCU may optimize supply of N in soil and increase adsorption of N in crops to improve fertilizer use efficiency and reduce environmental pollution. However, further field tests under contrasting soil types and crop rotations are required to confirm this potential use of LCU. Currently, the long-term LCU field tests are arranged to investigate the feasibility of LCU on enhancing crop productivity and mitigating N losses in cropland use, and the relevant results will be reported in our follow-up work after systematic scientific field experiments.

## Materials and Methods

### Materials

Samples of the upper 15-cm of paddy soil were collected from Yixing Base for Agri-Environment Research, Changshu National Agro–Ecosystem Observation and Research Station, Chinese Academy of Sciences (31°16′N, 119°54′E), near Taihu Lake. The soils are classified as Gleyi-Stagnic Anthrosols, and originate from Lacustrine parent material; samples comprised 8.3% sand, 81.5% silt, and 10.2% clay (v/v), with 15.4 g kg^−1^ organic C and 1.79 g kg^−1^ N, and cation exchange capacity and pH (H_2_O) of 11.8 cmol kg^−1^ and 5.6, respectively. Soil samples were air-dried and sieved through a 2-mm screen prior to treatment with LCA, LCU, and contrasting rates of N. Analytical reagent grade chemicals used in the experiments were supplied by Sinopharm Chemical Reagent Co., Ltd. (Shanghai, China).

### Samples characterization

Structure of the samples was characterized using X-ray diffraction (XRD) with a SmartLab diffractometer (Rigaku) equipped with a 9 kW rotating anode Cu source at 45 kV and 100 mA, from 5 to 50° with a scan rate of 0.2° s^−1^. We recorded infra-red spectra using a Nicolet iS10 FT-IR instrument (KBr disks) in the 400–500 cm^−1^ region, and solid state ^1^H NMR spectrum analysis was performed at 400 MHz using Hahn echo at room temperature at a spinning rate and contact time of 10 KHz and 2.5 ms, respectively; scan number was 1.2. Thermogravimetric (TG) analysis was carried out using a STA 409 instrument in dry air at a heating rate of 10 °C min^−1^. Morphology was tested using a field-emission scanning electron microscope (SEM) instrument (Hitachi S-4800), and specific surface area and pore diameter were obtained from N_2_-adsorption-desorption isotherms using multipoint BET and t-plot methods, where samples were outgassed at 300 °C to a vacuum of 10^−3^ Torr prior to measurement, and isotherms were obtained at liquid N temperature with a BEL SORP-MAX analyzer. Contact angle was measured using a contact-angle meter (DropMeter A-100P) at 25 °C. The chemical compositions of samples were obtained using ADVANT’XP X-ray fluorescence (XRF) spectrometer (ZSX Primus II), and the elemental analysis was performed on an elemental analyzer Vario EL cube. The concentrations of NH_4_^+^-N, NO_3_^−^-N, and total N (TN) in deionized water (or KCl) were analyzed using a continuous-flow N analyzer (Skalar San++ System, Netherlands).

### Hydrothermal treatment process

The original LCA (Henan Xin Lian Xin Fertilizer Co., Ltd., China) was treated with deionized water to improve its dispersion, and the resulting suspension (10 g L^−1^) was dried at 80 °C, following hydrothermal treatment at 180 °C for 2 h, with constant stirring, to achieve the final P-LCA powders. For the original LCU (Henan Xin Lian Xin Fertilizer Co., Ltd., China), the resulting suspension (3 g L^−1^) was dried at 60 °C, following hydrothermal treatment at 30 °C for 30 min, with constant stirring, to achieve the final P-LCU powders.

### Assessment of NH_3_ volatilization

We assessed NH_3_ volatilization according to previously reported methods^62–64^, where 2 mL of boric acid solution was placed with color reagent (dimethyl red-bromocresol green) in the center of a Petri dish, around which 10 g of soil (65% WHC, 30 °C) was added. Then, the N application rates of 80, 160, and 240 kg N ha^−1^ were added to three replicates of soil using a sample gun, before the Petri dish was covered and sealed (using Vaseline) and placed in an incubator for 23 d. Volatilized NH_3_ absorbed by boric acid was assessed at regular intervals using the acid-base titration method.

### Assessment of rapid N leaching

N leaching was measured using a previously published method^42^, where 30 g of dry sand (150–200 mesh) and 5 mL of deionized water to create humid conditions (relative humidity: 30%) were placed in a 50-mL centrifuge tube that had a 2-mm diameter hole at the base. Then, 1 g of LCU or 0.9 g of urea (2-cm diameter × 1-cm long cylinder shaped) was added to the tube and covered with 10 g of dry sand, prior to storage at 30 °C. We sprayed 50 mL of deionized water over the surface of the sand layer, and mass percent of N in the leachate and sand was calculated as: (mass of N in the leachate or sand/the mass of initial N) × 100.

### N leaching within a soil column

N leaching from the soil column was tested employing a earlier published method^21^, where 618 g of soil (65% WHC) was placed in a 30-cm tall soil column that was equally divided into top, middle, and bottom parts, to which urea or LCU with 80, 160, or 240 kg N ha^−1^ was added and stored at 30 °C for 110 d. Every 10 d, deionized water was added to the top of the column, and leachate was extracted from the base using a pump; soil WHC was maintained at a constant. The leachate was filtered through a 0.45-μm filter membrane and N concentration was quantified using a flow analyzer. After 110 d, about 10 g of soil was removed from each of the three layers of the soil column, to which 50 ml of KCl (2 mol L^−1^) solution was added, and concentration of extracted N in the filtrate was detected using a flow analyzer. TN in the soil column was calculated as sum of N in three layers.

## Supplementary information

Supplementary Information
