# Supplementary material for: Mitigation of ammonia volatilization and nitrate leaching via loss control urea triggered H-bond forces
Source: Sci Rep. 2019 Oct 22;9:15140. doi: 10.1038/s41598-019-51566-2 (PMC6805906; doi:10.1038/s41598-019-51566-2)
Supplement: Supplementary file 1 — Supplementary Information [file 41598_2019_51566_MOESM1_ESM.doc]

**Supplementary Materials**

**Mitigating ammonia volatilization and nitrate leaching via loss control urea triggered H-bond force**

Zhipan Ma^1^, Yanjun Yue^2^, Mengxi Feng^2^, Yushun Li^2^, Xue Ma^2^, Xu Zhao^1,*^, Shenqiang Wang^1^

^1^ State Key Laboratory of Soil and Sustainable Agriculture, Changshu National Agro-Ecosystem Observation and Research Station, Institute of Soil Science, Chinese Academy of Sciences, Nanjing 210008, China

^2^ Henan Xin Lian Xin Fertilizer Co., Ltd., Xinxiang 453700, China

*Corresponding author. Tel: +86-25-86881534; Fax: +86-25-86881028.

E-mail: zhaoxu@issas.ac.cn.

**Supplementary Figures:**


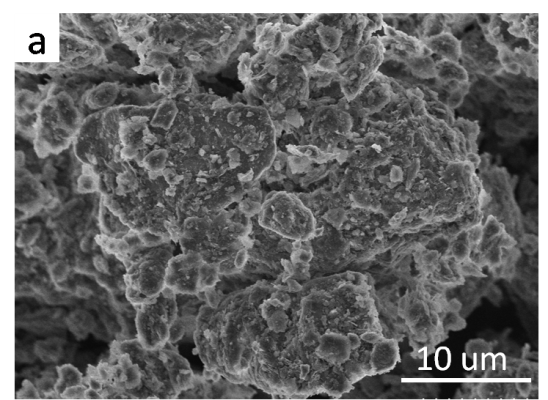

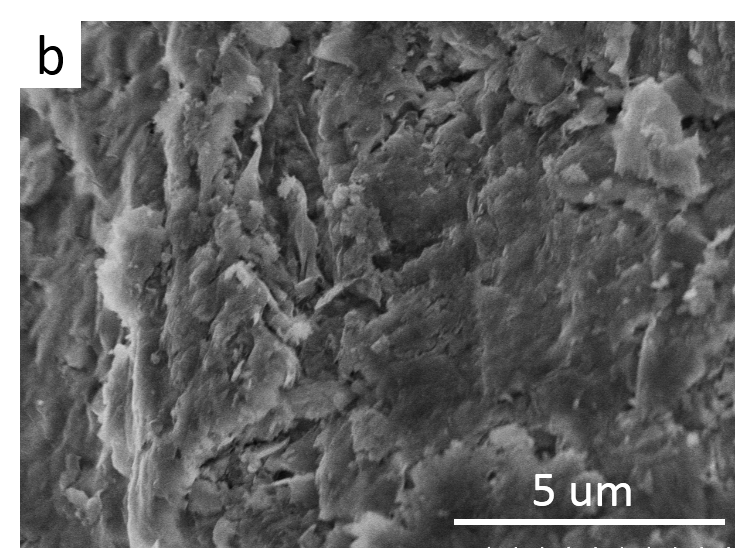

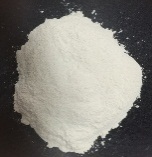

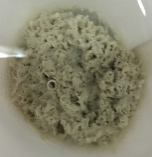


**Supplementary Figure S1.** SEM images of (a) loss control agent and (insert) sample, (b) pretreatment loss control agent and (insert) sample.

**Supplementary Figure S2.** Water retention capacity of loss control agent (LCA). Error bars indicate standard deviation of replicates (n = 3).

**Supplementary Figure S3.** Cumulative losses of NH_3_ volatilization from a 23-day soil incubation treated with urea or LCU at N rates of 0, 80, 160 and 240 kg N ha^-1^ under 30 °C and 65% soil water-holding capacity. Error bars indicate standard deviation of replicates (n = 3) and different letters indicate significantly difference at p≤0.05.

**
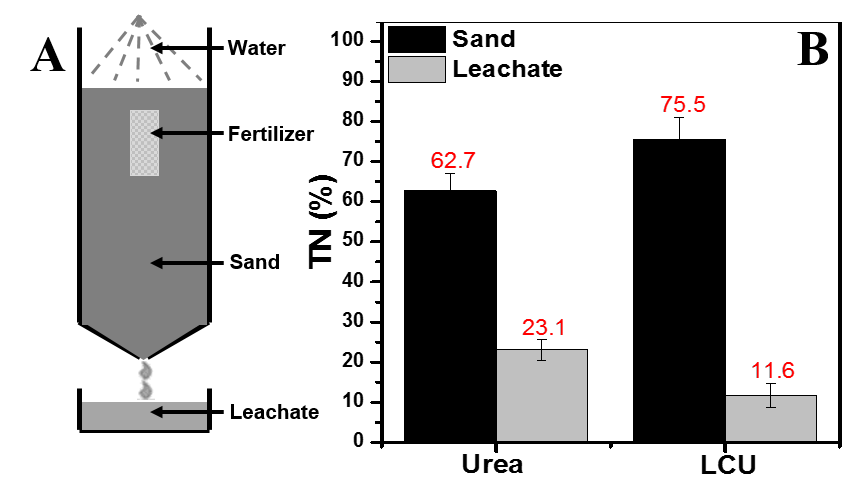
**

**Supplementary Figure S4.** (A) Schematic diagram of the rapid N leaching system and (B) mass percent of total N (TN) in leachate and sand for urea and loss control urea (LCU). The number in the picture refers to the mass percent of TN in sand and leachate for urea or LCU, respectively. Error bars indicate standard deviation of replicates (n = 3).

**Supplementary Figure S5.** Mineral nitrogen content of (A) leachate and (B) sand retention in rapid N leaching test. Error bars indicate standard deviation of replicates (n = 3).

**Supplementary Figure S6.** Measured amounts of (A) leaching and (B) retention of fertilizer nitrogen (N) at the end of experiment from 110 days incubation. Error bars indicate standard deviation of replicates (n = 3).

**Supplementary Figure S7.** Measured amounts of retention of fertilizer nitrogen (N) respectively within the top, middle and bottom of the soil column at the end of experiment from 110 days incubation. Error bars indicate standard deviation of replicates (n = 3).

**Supplementary Table**

**Supplementary Table S1. Composition analyses of LCU detected by XRF.**

| Sample | C (wt.%) | N (wt.%) | **Urea (wt.%)^a^** |
| --- | --- | --- | --- |
| LCU | 19 | 44 | 94.6 |

^a^ **Urea (wt.%)=****[**C (wt.%)/(12/60)] or **[**N (wt.%)/(28/60)], the value of 94.6% is the average.

**Supplementary Table S2. Brief remarks of all the samples prepared or examined.**

| Samples | Brief remarks |
| --- | --- |
| U | urea |
| LCA | loss control agent |
| LCU | loss control urea (U: LCA=94:6) |
| P-LCA | pretreatment loss control agent |
| P-LCU | pretreatment loss control urea |
